# 

**Published:** 1874-04

**Authors:** 


### CONTENTS:

### ORIGINAL COMMUNICATIONS.

ARTICLE I.—Professional Excellence. By W. W. H. Thackston, M D., D. D. S. .......... 529

ARTICLE II.—Fragmentary Clippings. By S. P. Cutler, M. D., D. D. S. .......... 533

ARTICLE III.—Class Address. By John W. Farmer, M. D., D. D. S. .......... 543

### SELECTED ARTICLES.

ARTICLE IV.—Tobacco. By J. W. Mason, M. D. .......... 547

ARTICLE V.—The Recent Death in a Dentist's Chair. By J. F. Babcock, D. D. S. .......... 552

ARTICLE VI.—Pepsin. By T. R. Edes, M. D. .......... 556

ARTICLE VII.—Preparation of Cavities. By C. E. Francis, D. D. S. .......... 561

ARTICLE VIII.—On Saponin as a Local Anæsthetic .......... 564

ARTICLE IX.—Case of Excision of Left Superior Maxillary and Molar Bones and one-half of the Nose. By M. Metivier, M. D. .......... 565

### EDITORIAL, ETC.

Dentistry .......... 568

Dentistry by the Hour .......... 569

Reaching Pulp Canals .......... 570

Salivation Produced by Mixing Amalgams in the Hands .......... 571

### BIBLIOGRAPHICAL.

Dunglison's Dictionary of Medical Science. Revised Edition .......... 571

Transactions of American Dental Association for 1873 .......... 572

### MONTHLY SUMMARY.

The Siamese Twins .......... 572

Needle Swallowed by a Child, Removed Six Months afterwards from the Thigh .......... 573

A New way of Gargling .......... 574

A New Operation for Cleft Palate .......... 574

New Source of India Rubber .......... 575

Ergot in Epistaxis .......... 576

Cure for Baldness .......... 576

